# Efficacy of Folic Acid Supplementation in Autistic Children Participating in Structured Teaching: An Open-Label Trial

**DOI:** 10.3390/nu8060337

**Published:** 2016-06-07

**Authors:** Caihong Sun, Mingyang Zou, Dong Zhao, Wei Xia, Lijie Wu

**Affiliations:** 1 Department of Children’s and Adolescent Health, Public Health College of Harbin Medical University, Harbin 150081, China; suncaihong2003@163.com (C.S.); mingyangshine@sina.com (M.Z.); 2 Zhejiang Provincial Center For Disease Prevention and Control, Hangzhou 310009, China; dzhao@cdc.zj.cn

**Keywords:** autism, folic acid intervention, structured teaching, homocysteine, glutathione redox status

## Abstract

Autism spectrum disorders (ASD) are recognized as a major public health issue. Here, we evaluated the effects of folic acid intervention on methylation cycles and oxidative stress in autistic children enrolled in structured teaching. Sixty-six autistic children enrolled in this open-label trial and participated in three months of structured teaching. Forty-four children were treated with 400 μg folic acid (two times/daily) for a period of three months during their structured teaching (intervention group), while the remaining 22 children were not given any supplement for the duration of the study (control group). The Autism Treatment Evaluation Checklist (ATEC) and Psychoeducational Profile-third edition (PEP-3) were measured at the beginning and end of the treatment period. Folic acid, homocysteine, and glutathione metabolism in plasma were measured before and after treatment in 29 autistic children randomly selected from the intervention group and were compared with 29 age-matched unaffected children (typical developmental group). The results illustrated folic acid intervention improved autism symptoms towards sociability, cognitive verbal/preverbal, receptive language, and affective expression and communication. Furthermore, this treatment also improved the concentrations of folic acid, homocysteine, and normalized glutathione redox metabolism. Folic acid supplementation may have a certain role in the treatment of children with autism.

## 1. Introduction

The autism spectrum disorders (ASD) are a complex group of neurodevelopmental disorders characterized by abnormal social interaction, impaired communication, and repetitive stereotypic behaviors. ASD affect one in 45 births in the United States, according to a new government estimate of the condition’s prevalence in 2014 [1]. Over the last decade, increasing attention has been given to the pathophysiological abnormalities observed in ASD, including immune dysregulation, mitochondrial dysfunction, and metabolic abnormalities. Case-control studies have uncovered that the metabolic profile of children diagnosed with ASD was abnormal compared with that of unaffected control children [2,3,4,5]. Briefly, folate, homocysteine (Hcy) and glutathione metabolism were abnormal in many autistic children.

**Figure 1:**
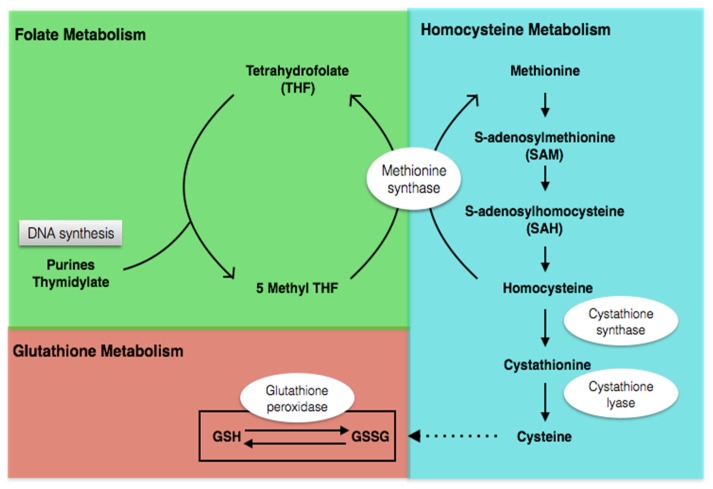
Folate, homocysteine, and glutathione metabolism.

Metabolism of folate and Hcy are important determinants regulating one-carbon metabolism and metabolic balance between the remethylation and transsulfuration pathways (Figure 1). It has been shown that abnormal folic acid disturbed *de novo* purine and pyrimidine synthesis as well as the remethylation of Hcy into methionine [6]. Subsequently, abnormal methionine affected the conversion of methionine into S-adenosylmethionine (SAM), a primary methyl donor for most cellular methytransferase reactions, including the methylation of DNA, RNA, proteins, phospholipids, and neurotransmitters [3]. Alongside these, folate was linked to methylation and epigenetic changes. Hcy is normally metabolized via two biochemical pathways, the transsulfuration pathway and the remethylation pathways, the latter converting Hcy back to methionine. The transsulfuration pathway, which is closely linked to the folate-methionine cycle, involves conversion of Hcy to cystathionine and ultimately to cysteine in two vitamin B6 dependent reactions catalyzed by cystathionine-beta-synthase and cystathionase (CBS), respectively [6]. Therefore, the synthesis of the primary low-molecular cellular antioxidant glutathione is interdependently linked to the folate pathway, thus any aberrant disruptions in folate metabolism can result in potentially deleterious effects as a result of an imbalance in the cellular redox state.

Humans are not able to synthesize folic acid and thus are dependent on dietary sources. Evidence has shown that autistic children are picky eaters and/or have gastrointestinal problems that result in inadequate dietary intake and micronutrient deficiencies [7]. Folic acid deficiency affects cellular methylation and indirectly unbalances cellular redox homeostasis. In addition, experimental studies have shown that methylation impairment and oxidative stress may be contributing factors to autism pathology [8,9,10]. Finally, there is an association between the observed decrease in glutathione redox status and methylation capacity and micronutrient deficiencies in many autistic children.

Several recent research studies also reported that folic acid could notably impact cognitive funtion or autistic-like behaviors [11,12,13,14]. However, there was no study referring to association between structured teaching and folic acid treatment. In the present study, folic acid was given to autistic children participating in structured teaching in order to determine whether or not this intervention could be effective at improving core symptoms of autism during structured teaching. At the same time, methylation capacity and the redox status in a cohort of autistic children were also tested. The results of this study may help to determine the underlying causes of autism in a subgroup of children and tailor new methods for treating autistic children with this specific biochemical alteration.

## 2. Materials and Methods

### 2.1. Participants

The study was conducted between March 2011 and June 2012 in the Child Development and Behavior Research Center of Harbin Medical University, Harbin, China. All enrolled children (83 autistic children and 29 age-matched unaffected children) were Chinese Han. The present study was approved by the Institutional Review Board of Harbin Medical University for Medical Sciences (No. 2010069) and written informed consent was obtained from all parents prior to the study.

In part I of the study, 83 children with ASD who participated in structured teaching completed an open-label trial and were divided into an intervention group, supplemented with the folic acid for a period of three months, and a control group, which was not given any supplementation. Inclusion criteria were a diagnosis of ASD, which was made by a specialist clinician and confirmed by the Diagnostic and Statistical Manual of Mental Disorders, fourth edition (DSM-IV) diagnostic criteria. Exclusion criteria: children with Asperger’s or pervasive developmental disorders not otherwise specified, genetic disorders, chronic seizures, severe gastrointestinal symptoms, recent infection, or those who recently used high-dose vitamins or mineral supplements were ruled out.

Seventeen children dropped out of the study due to inconsecutive structured teaching and/or folic acid intervention or were lost for a variety of unspecified reasons (*n* = 11 in the intervention group; *n* = 6 in the control group). The 66 remaining children who completed the study consisted of 37 boys (77%) and seven girls (23%) (mean age: 57.23 ± 15.06 months) in the intervention group and 17 boys (77%) and five girls (23%) (mean age: 51.75 ± 12.72 months) in the control group.

In part II of the study, 34 autistic children from the intervention group gave consent to have blood drawn before and after the experiment and of those, 29 children (24 boys and five girls, mean age: 52.00 ± 13.13 months) completed the three-month folic acid intervention. Twenty-nine healthy children were selected from the Community Health Care Center, Harbin, China, according to the proportion of 1:1 in gender, age, socioeconomic status, typical developmental stage, and no previous history of developmental delay or neurologic disorders (24 boys and five girls, mean age: 57.36 ± 10.30 months). 

**Figure 2:**
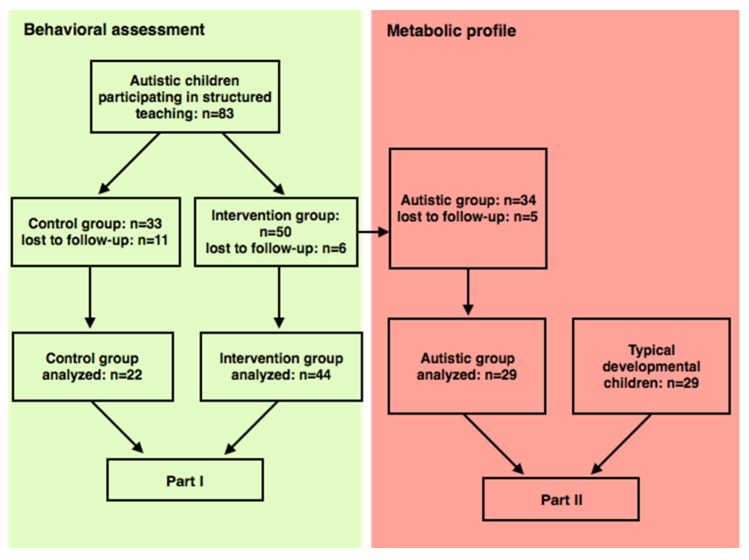
Flowchart of study design and patient follow-up.

A flowchart of the intervention study design is presented in Figure 2.

### 2.2. Nutritional Supplements

According to the tolerable upper intake level designated for children by China and the nature of the synthetic folic acid, 44 autistic children in the intervention group were treated orally with twice daily 400 µg (a total of 800 µg/day) supplementation of folic acid for three months. Folic acid tablets (400 µg) were obtained from Beijing North Pharmaceutical Co. Ltd, Beijing, China. All parents were given a demonstration and instructions for the intervention, and were also asked to record feedback information during the intervention. A further 22 autistic children in the control group participated in structured teaching without any vitamin/mineral supplementation.

### 2.3. Structured Teaching

The emphasis on improving the outcomes of children with ASD has generally been on early intervention, usually relating to the treatment and education of autistic and related communication handicapped children (TEACCH). This intervention program was developed to support people of all ages with ASD in order to effectively reduce challenging behavior and improve communication, sensorimotor skills, and independence [15]. In addition, the philosophy of TEACCH includes helping the affected family understand autism and also focuses on an individual’s specific needs. Structured teaching, the major component of the TEACCH program, was established for each individual with autism on the basis of a Psychoeducational Profile, third edition (PEP-3), assessment of ability. In the present study, experienced nursery school teachers, who were trained in the TEACCH methodology, taught the three month structured course. The four major components of structured teaching are physical structure (the organization of the classroom), schedules (visual information depicting where/when/what the activity will be), work systems (visual information informing a student what to do while in a work or play area), and task organization (visually clear information on what the learning task is about).

### 2.4. Evaluation

Four formal assessment tools were used in this study: autism behavior checklist (ABC), childhood autism rating scale (CARS), autism treatment evaluation checklist (ATEC; a parental report), and PEP-3 (teacher observations), all of which were administered by a trained nurse at the baseline visit and again at the end of the treatment period.

ABC is a 57-item scale for characterizing autism behaviorisms. A cut-off total score of >67 was used for indicating children with a high probability of autism, while a total score between 53 and 67 indicated questionable autism.

CARS is a 15-item behavioral rating scale developed to identify autism as well as quantitatively describe the severity of the disorder [16].

ATEC is used to evaluate the effectiveness of treatments for autistic patients [17]. The assessment is reported by a caregiver and yields scores in four areas which are characteristically problematic for individuals with ASD: communication, sociability, sensory, and health. High scores indicate more problems within each area.

PEP-3 [18,19], specifically designed for children with ASD, was used to estimate the development of communication and motor skills as well as maladaptive behaviors. The PEP-3 was proposed as an assessment tool to evaluate the treatment effects of the TEACCH. The PEP-3 includes a performance aspect, which is administered by direct testing and observation, and a caregiver report. The performance part is composed of 10 subscales: cognitive verbal/preverbal (CVP), expressive language (EL), receptive language (RL), fine motor (FM), gross motor (GM), visual motor imitation (VMI), affective expression (AE), social reciprocity (SR), characteristic motor behaviors (CMB), and characteristic verbal behaviors (CVB). These 10 subscales are combined into three composites: communication (CVP, EL, and RL), motor (FM, GM, and VMI), and maladaptive behaviors (AE, SR, CMB, and CVB). The caregiver report is composed of three subscales: problem behaviors (PB), personal self-care (PSC), and adaptive behaviors (AB). Raw scores of the subscales can be transformed into developmental age and percentile rank for each subscale and composite.

### 2.5. Sample Treatment and Metabolite Analysis

Fasting blood samples were collected into EDTA-evacuated tubes and immediately chilled on ice before centrifuging at 3000 r/min for 10 min at 4 °C. To prevent metabolite interconversion, the ice-cold samples were centrifuged within 15 min of the blood collection and the plasma stored at −20 °C until high-pressure liquid chromatography (HPLC) quantification within two weeks after receipt. 

### 2.6. Statistics

The data were prospectively collected and analyzed using EpiData 3.02 (EpiData Association, Odense, Denmark) and SPSS 17.0 (SPSS Inc., Chicago, IL, USA) software. For the descriptive part of the experiment, we computed the means and standard deviation of the demographic and outcome variables (independent *t* test, two-tailed). We used repeated measures analysis of variance (ANOVA) to test the effect of folic acid intervention and structured teaching on the ATEC and PEP-3 scores. For all analyses, significance was set at *p* < 0.05. Statistical differences in plasma metabolites before and after intervention and between controls were determined by Student’s paired *t* test.

## 3. Results

### 3.1. Clinical Features

In part I of the study, no significant difference emerged in terms of age, sex, the ABC, and CARS between the participants assigned to each group (*p* > 0.05). There were also no significant differences in the four ATEC subscales, total ATEC score, and 16 PEP-3 domains (*p* > 0.05; Table 1).

### 3.2. Behavior Change over the Intervention Period

Following structured training with or without folic acid treatment for three months, repeated measures ANOVA did not show a significant intervention effect or interaction between time/structured teaching and intervention in the ABC and the CARS scores. However, there were significant time/structured teaching effects in both the ABC and the CARS scores (*p* < 0.05).

Repeated measures ANOVA analyses for the ATEC scale revealed significant intervention × time/structured teaching interactions in the ATEC sociability subscale (*p* < 0.05). Analyses for the other three ATEC subscales and ATEC total score yielded statistically significant main effects for time/structured teaching (*p* < 0.05), however, there were no significant main effects for intervention.

Repeated measures ANOVA analyses for the PEP-3 data showed significant intervention × time/structured teaching interactions in CVP, RL, AE, and communication (*p* < 0.05). Analyses with the EL, FM, GM, VMI, SR, CVB, AB, and motor and maladaptive behaviors domains yielded statistically significant main effects for time/structured teaching (*p* < 0.05), however, there were no significant main effects in the CMB, PB, and PSC domains. Significant main effects for intervention did not emerge in EL, FM, GM, VMI, SR, CVB, CMB, PB, PSC, AB, or motor and maladaptive behaviors domains (Table 2).

### 3.3. Metabolic Profile

Baseline: The results indicated that there were significant differences in the baseline concentrations of the folic acid, Hcy, total reduced glutathione (tGSH), oxidized glutathione disulfide (GSSG), and the ratio of tGSH/GSSG between autistic children and their matched healthy controls (*p* < 0.05), however, vitamin B12 concentrations were not significantly different (*p* > 0.05).

Endpoint: Vitamin B12 concentrations did not differ between pre-treatment and post-treatment group (*p* > 0.05). The three-month intervention significantly increased plasma folic acid in the post-treatment compared to pre-treatment group, and compared to the controls (*p* < 0.05). The mean concentration of Hcy significantly decreased after intervention and was below those in controls (*p* < 0.05). The tGSH level and tGSH/GSSG ratio were significantly higher after intervention and were not statistically different compared with those in the control group. Mean concentration of GSSG significantly decreased after intervention (*p* < 0.05), and remained above the mean concentration in the control group, although this was not significant (Table 3).

**Figure 3:**
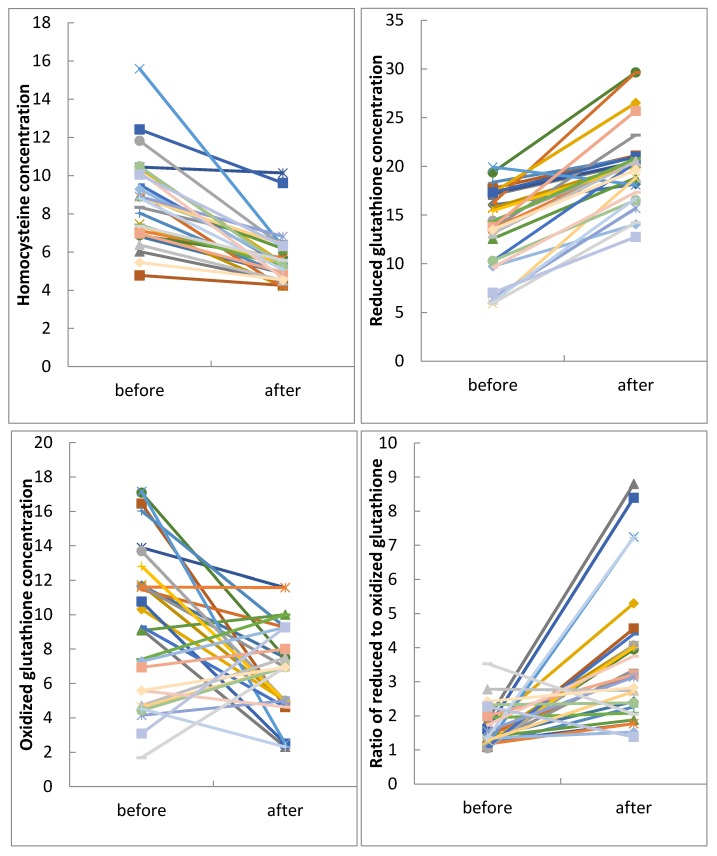
Scatter plots of individual data for plasma Hcy, tGSH, GSSG levels and tGSH/GSSG ratios from 29 autistic children before and after three months of folic acid treatment.

In Figure 3, scatter plots showing the distribution of the individual data points in the autistic group before and after intervention are presented for Hcy, tGSH, GSSG, and the tGSH/GSSG ratio (Figure 3).

## 4. Discussion

This intervention trial was undertaken to determine whether nutritional support with folic acid could improve ASD associated behavior as measured by the ATEC and PEP-3 in autistic children, and at the same time, improve the metabolic profile of these children. In addition, according to parents, there were no side effects reported in this study. This indicates folic acid treatment is safe and warrants further study as an intervention.

### 4.1. Behavioral Improvements with Intervention

In part I of this study, the primary outcome revealed that folic acid intervention had an auxiliary therapeutic action on improvement of sociability, cognitive verbal/preverbal, receptive language, affective expression, and communication for the autistic children participating in structured teaching. To the best of our knowledge, this is the first combined folic acid intervention and structured teaching test, and, overall, significant improvement was noted in all subtests of the ATEC and most subtests of PEP-3 (except characteristic motor behavior, problem behavior, and personal self-care) during the three months of structured teaching. The TEACCH approach is used worldwide, with reported benefits for the individuals using it. There is a large body of scientific literature demonstrating that the TEACCH approach aids autistic children with improving the main impairments associated with this disease, such as personal independence, social abilities, and functional communication [15,20]. The results of the present study support these data and are in accordance with previous reports. Kvestad *et al*. showed supplementation with folic acid and/or vitamin B12 for six months could improve gross motor and problem solving skills in healthy children aged 30 months to six years [11]. Moretti *et al*. found that after continuous folinic acid supplementation for a year, autistic children with low levels of 5-methyltetrahydrofolate in their cerebrospinal fluid showed improvement in neural development and in cognitive and behavioral ability [21]. Additional studies have reported that following orally administered leucovorin, autistic children showed partial improvements in communication, social interaction, attention, stereotypical behavior, and cognition [10,22,23]. A large clinical trial showed supplementation with 20 vitamins and 14 minerals improved symptoms associated with autism, including receptive language, hyperactivity, and tantrums [24].

High concentrations of Hcy resulted from folate deficiency, as an agonist of glutamatergic receptors can alter glutamatergic transmission in certain brain areas involved in communication skills, and information has emerged regarding a connection between communication deficit and Hcy metabolism [25,26]. Although the biological mechanisms linking folic acid supplementation to improvement in ASD symptoms are not known, the aforementioned reports have indicated folic acid may have a certain role to play in the treatment of children with autism. 

### 4.2. Metabolic Profile before and after Intervention

Abnormal folate metabolism and low glutathione concentrations have been reported in other neurologic disorders [26,27,28,29], including Alzheimer disease, Parkinson’s disease, schizophrenia, and Down syndrome. As methylation impairment and oxidative stress were assessed in the above studies, we wished to determine whether these existed in children with ASD and therefore measured the concentrations of folic acid, Hcy, and glutathione redox metabolites in children with or without ASD.

Folate and vitamin B12 are independent and essential components of the one-carbon metabolic pathway. Deficiency in vitamin B12, which is a coenzyme for methionine synthase, leads to a functional deficiency in folate. In the current study, there was no significant difference in the concentration of vitamin B12 between pre-treatment and controls, and between pre-treatment and post-treatment, at the same time the concentration of vitamin B12 was always within the normal range. Therefore the results of this study concluded that there was no association between vitamin B12 and abnormal metabolism.

#### 4.2.1. Metabolic Profile before Intervention

Folic acid plays a key role during neural development and acts as a coenzyme in the one-carbon metabolic pathway which is utilized in a number of processes including DNA synthesis, cell proliferation, and immune function [3,30]. Folate deficiency affects normal brain development through a variety of mechanisms and the deficiency of folic acid during pregnancy has been reported as a risk for offspring developing ASD [31]. Interestingly, we found lower folic acid plasma concentrations in children with ASD than their controls in the current study. However, Pasca *et al.* did not show any difference in folate levels between ASD patients and controls in Caucasian populations [32]; nonetheless, other studies have proven to support our results regarding low folate levels in ASD patients [4,5].

It has been shown that Hcy concentrations could also be regulated by genetic and environmental factors influencing folate. Impaired transfer of methyl groups via the methionine cycle leads to plasma hyperhomocysteinemia. Elevated concentrations of Hcy, which are characteristic of hyperhomocysteinemia, have been shown to be the most prominent biochemical sign of functional folate and vitamin B12 insufficiency. Moreover, hyperhomocysteinemia can cause brain dysfunction via oxidative damage and abnormal DNA methylation [33]. The results of the present study are in line with previous reports regarding the findings of increased Hcy concentrations in biological fluids in children/adolescents with ASD compared to age-matched healthy controls [24,34,35]. There is an unexpected result reported by James that the congruous level of Hcy existed in autistic children in comparison with the control group, nevertheless, 16 out of 20 patients were taking 400 µg folic acid supplements prior to the beginning of the study. Such a treatment decreased the level of Hcy in the plasma of autistic children and the control group [36]. Therefore we conclude that high Hcy and low folate levels may be useful biomarkers for the early diagnosis of ASD.

In this study, autistic children had significantly lower tGSH and increased GSSG disulfide, resulting in a significantly lower tGSH/GSSG ratio compared with healthy controls. These abnormal glutathione metabolite levels prompted us to hypothesize that autistic children of Chinese Han descent had increased oxidative stress levels compared to normal children of the same background. Indeed, a previous study examined postmortem ASD brains and found evidence of increased oxidative stress levels [8,9]. Several studies have also reported decreased glutathione redox ratios in patients with ASD [2,10]. Taken together with the current findings, these data support the hypothesis that a shift in the glutathione redox ratio and redox imbalance may contribute to the etiology of autism [9,37]. It must be noted, however, the underlying mechanisms associated with increased oxidative stress in children with ASD are complex and cannot be explained by abnormalities in a single pathway. Thus, more extensive research is needed to tease out the complex mechanisms associated with this phenomenon.

#### 4.2.2. Metabolic Profile after Intervention

The three-month folic acid treatment in the present study was successful in increasing the mean concentrations of folic acid, tGSH, and the ratio of tGSH/GSSG, and reducing the mean concentrations of Hcy and GSSG. The observed change indicated that folic acid intervention stimulated remethylation of Hcy into methionine and simultaneously diverted Hcy to the transsulfuration pathway. The results we found were consistent with other studies. Aruna *et al*. demonstrated Hcy negatively correlated with folic acid levels [38]. The observed higher red blood cell folate status and lower Hcy were the direct result of folic acid intake [39]. A double-blind placebo-controlled study found an increase in the concentration of GSH and the ratio of GSH/GSSG in plasma following oral vitamin/mineral supplementation [24]. An open label-trial indicated methylcobalamin and folinic acid treatment improved glutathione redox status [40] and several lines of evidence support the notion that glutathione metabolism can be improved by vitamin, mineral, antioxidant, and B vitamin supplementation.

Glutathione status is an accurate indicator of cell functionality and viability. The ratio of GSH/GSSG (antioxidant capacity) ensures the reducing intracellular environment that is required for normal immunity function; detoxification capacity, redox-sensitive enzyme activity, and membrane redox signaling. A shift in the glutathione redox ratio towards the oxidized state may lead to decreased cell proliferation, DNA damage, and increased apoptosis [2]. Such a shift could potentially affect neurological development in the early stages of life.

We hypothesize that folic acid supplementation could elevate antioxidant glutathione through a variety of mechanisms including: (1) As Hcy can partially induce the formation of the powerful oxidant peroxynitrite, consumption of antioxidative glutathione compensated for potential oxidative damage from excess Hcy [41]. This consumption effect wore off when Hcy decreased with the folic acid supplementation; (2) Hcy may negatively affect glutathione peroxidase activity and thus transcription of glutathione. Therefore, the reduced Hcy levels observed in this study could have led to an upregulation of the enzyme, thereby increasing glutathione synthesis [33]; (3) CBS can be activated allosterically by SAM and for this reason, when SAM levels increase due to folic acid supplementation, cystathionine formation is increased [6]. Therefore, the observed reduction in Hcy may have resulted in a concomitant increase in plasma cysteine, the rate-limiting amino acid for glutathione synthesis. A folic acid supplement provides not only methyl groups for the synthesis of methionine but also secondary precursors for subsequent downstream glutathione synthesis.

Folinic acid can be absorbed rapidly without dihydrofolate reductase (DHFR), whereas folic acid, the synthetic form of folate, should be converted by DHFR to a bioactive form before entering folate metabolism [36]. Nevertheless, this does not mean that it is inappropriate to take folic acid. There is evidence that folic acid could be absorbed and utilized by humans within a tolerable upper intake level (1000 µg/day), and DHFR has sufficient capacity in humans to efficiently metabolize normal doses of folic acid [42,43,44,45]. Furthermore, folic acid is more readily available than folinic acid in China.

### 4.3. Limitations

There are a couple of potential weaknesses of this study. First, as this study was an open-label and nonrandomized study, we could not clearly determine whether the metabolism change over time in the children participating in the study was due to folic acid treatment specifically, or to placebo effects. Second, due to the limited size of our sample, statistical power was low and further analysis will be required to clarify these findings, and we cannot generalize the results for all children with ASD. Given the lack of available specific pharmacological therapies for ASD and the clinical heterogeneity of the disease, current research suggests more detailed and specific studies will be required to evaluate the pathophysiology associated with ASD. Clearly, a double-blind placebo-controlled trial should be performed to eliminate the potential bias associated with an open-label test.

## 5. Conclusions

This study demonstrates that a three-month folic acid intervention in autistic children participating in structured teaching significantly improved symptoms of autism as measured by ATEC in regards to sociability, and PEP-3 in regards to cognitive verbal/preverbal, receptive language, affective expression, and communication. This treatment also altered the concentrations of folic acid, Hcy, and normalized glutathione redox metabolism. These results unravel that children with ASD—or, at the very least, a subset of children with ASD—could benefit from this simple and safe nutritional supplementation.

## Figures and Tables

**Table 1:** Clinical features in the intervention and control groups, respectively.

Features	Intervention (*n* = 44)	Control (*n* = 22)
Age (month)	57.23 ± 15.06	51.75 ± 12.72
Sex (male:female)	37:7	17:5
ABC	54.55 ± 26.58	67.59 ± 27.60
CARS	33.86 ± 7.08	33.41 ± 6.04
ATEC		
Communication	9.93 ± 7.02	11.09 ± 6.91
Sociability	8.93 ± 5.84	10.09 ± 5.30
Sensory and cognitive awareness	12.86 ± 7.19	15.14 ± 7.03
Health behavioral problem	16.95 ± 7.69	21.05 ± 9.81
Total	48.68 ± 21.43	57.36 ± 20.38
PEP-3		
CVP	12.00 ± 3.00	12.95 ± 2.38
EL	11.41 ± 3.41	11.91 ± 2.86
RL	12.36 ± 2.42	13.32 ± 1.96
FM	11.80 ± 1.79	11.91 ± 2.00
GM	12.07 ± 1.78	12.36 ± 1.92
VMI	11.55 ± 2.17	12.00 ± 2.16
AE	11.80 ± 2.06	10.95 ± 2.30
SR	10.36 ± 2.08	10.50 ± 1.87
CMB	11.98 ± 2.39	11.95 ± 2.30
CVB	8.00 ± 3.58	9.05 ± 2.90
Communication	35.77 ± 8.21	38.18 ± 6.71
Motor	35.41 ± 5.18	36.27 ± 5.54
Maladaptive behavior	42.14 ± 7.84	42.45 ± 7.31
PB	8.25 ± 2.36	8.27 ± 2.39
PSC	11.70 ± 2.41	12.09 ± 2.27
AB	11.14 ± 2.36	11.36 ± 2.63

Abbreviations: ABC, autism behavior checklist; CARS, childhood autism rating scale; ATEC, autism treatment evaluation checklist; PEP-3, Psychoeducational Profile, third edition; CVP, cognitive verbal/preverbal; EL, expressive language; RL, receptive language; FM, fine motor; GM, gross motor; VMI, visual motor imitation; AE, affective expression; SR, social reciprocity; CMB, characteristic motor behaviors; CVB, characteristic verbal behaviors; PB, problem behaviors; PSC, personal self-care; AB, adaptive behaviors.

**Table 2:** Scores from ATEC and PEP-3 at baseline and after the three-month folic acid intervention in the intervention and control groups, respectively.

Scales	Point	Intervention (*n* = 44)	Control (*n* = 22)	*p* 1	*p* 2	*p* 3
ABC	Baseline	54.55 ± 26.58	67.59 ± 27.60	0.424	<0.001 *	0.080
	Endpoint	39.40 ± 26.73	46.18 ± 22.71			
CARS	Baseline	33.86 ± 7.08	33.41 ± 6.04	0.331	0.001 *	0.684
	Endpoint	29.34 ± 5.52	30.82 ± 5.06			
ATEC						
communication	Baseline	9.93 ± 7.02	11.09 ± 6.91	0.583	<0.001 *	0.354
	Endpoint	6.84 ± 5.65	8.64 ± 6.29			
sociability	Baseline	8.93 ± 5.84	10.09 ± 5.30	0.024 *	0.002	0.020
	Endpoint	5.23 ± 3.72	9.45 ± 5.12			
sensory and cognitive awareness	Baseline	12.86 ± 7.19	15.14 ± 7.03	0.349	<0.001 *	0.321
	Endpoint	11.36 ± 7.15	12.50 ± 5.71			
health behavioral problem	Baseline	16.95 ± 7.69	21.05 ± 9.81	0.542	<0.001 *	0.059
	Endpoint	12.86 ± 7.00	15.77 ± 7.79			
total	Baseline	48.68 ± 21.43	57.36 ± 20.38	0.719	<0.001 *	0.052
	Endpoint	36.30 ± 17.49	46.36 ± 18.56			
PEP-3						
CVP	Baseline	12.00 ± 3.00	12.95 ± 2.38	0.020 *	<0.001	0.593
	Endpoint	14.20 ± 1.97	13.91 ± 2.79			
EL	Baseline	11.41 ± 3.41	11.91 ± 2.86	0.451	<0.001 *	0.715
	Endpoint	13.07 ± 3.19	13.14 ± 2.87			
RL	Baseline	12.36 ± 2.42	13.32 ± 1.96	0.027 *	<0.001	0.313
	Endpoint	13.75 ± 1.40	13.73 ± 1.75			
FM	Baseline	11.80 ± 1.79	11.91 ± 2.00	0.328	<0.001 *	0.447
	Endpoint	12.48 ± 1.68	13.00 ± 1.75			
GM	Baseline	12.07 ± 1.78	12.36 ± 1.92	0.954	0.001 *	0.954
	Endpoint	12.77 ± 0.99	13.09 ± 1.31			
VMI	Baseline	11.55 ± 2.17	12.00 ± 2.16	0.860	0.010 *	0.349
	Endpoint	12.27 ± 1.48	13.64 ± 1.97			
AE	Baseline	11.80 ± 2.06	10.95 ± 2.30	0.047 *	0.012	0.674
	Endpoint	11.98 ± 2.25	12.45 ± 1.26			
SR	Baseline	10.36 ± 2.08	10.50 ± 1.87	0.717	<0.001 *	0.586
	Endpoint	11.30 ± 2.09	11.64 ± 1.65			
CMB	Baseline	11.98 ± 2.39	11.95 ± 2.30	0.149	0.628	0.404
	Endpoint	11.66 ± 2.58	12.59 ± 2.26			
CVB	Baseline	8.00 ± 3.58	9.05 ± 2.90	0.837	0.001 *	0.218
	Endpoint	9.64 ± 3.72	10.50 ± 2.65			
Communication	Baseline	35.77 ± 8.21	38.18 ± 6.71	0.037 *	<0.001	0.536
	Endpoint	41.02 ± 5.91	40.77 ± 7.04			
Motor	Baseline	35.41 ± 5.18	36.27 ± 5.54	0.749	<0.001 *	0.359
	Endpoint	37.52 ± 3.79	38.73 ± 4.69			
Maladaptive behaviors	Baseline	42.14 ± 7.84	42.45 ± 7.31	0.285	0.001 *	0.433
	Endpoint	44.57 ± 9.59	47.18 ± 6.53			
PB	Baseline	8.25 ± 2.36	8.27 ± 2.39	0.936	0.076	0.983
	Endpoint	9.05 ± 2.90	9.00 ± 2.65			
PSC	Baseline	11.70 ± 2.41	12.09 ± 2.27	0.612	0.131	0.636
	Endpoint	12.34 ± 1.70	12.41 ± 2.48			
AB	Baseline	11.14 ± 2.36	11.36 ± 2.63	0.202	0.010 *	0.786
	Endpoint	12.32 ± 2.59	11.77 ± 2.51			

*P* 1: intervention × time/structured teaching interactions; *p* 2: main effect for time/structured teaching; *p* 3: main effect for intervention; significant differences when compared between intervention and control indicated by *.

**Table 3:** Mean plasma metabolite levels (mean ± SD) in age-matched control children and autistic children before and after the three-month folic acid treatment.

Metabolite Levels	Typical Group (*n* = 29)	Autistic Group (*n* = 29)		*p* Value *
		Pre-Treatment	Post-Treatment	
VitB12 (pmol/mL)	523.30 ± 186.51	555.24 ± 249.00	544.17 ± 213.57	0.723
Folic acid (nmol/L)	28.60 ± 8.67	21.60 ± 9.09 ^a^	64.5 ± 14.45 ^a^	<0.001 *
Hcy (µmol/L)	7.80 ± 1.13	8.80 ± 2.29 ^a^	5.65 ± 1.39 ^a^	<0.001 *
tGSH (µmol/L)	17.84 ± 5.03	13.37 ± 4.26 ^a^	20.07 ± 4.03	<0.001 *
GSSG (µmol/L)	5.79 ± 2.81	9.44 ± 4.45 ^a^	6.68 ± 2.68	0.009 *
tGSH/GSSG	4.26 ± 3.25	1.61 ± 0.59 ^a^	3.67 ± 2.01	<0.001 *

* Pre- and post-treatment comparison; a: significantly different from typical developmental children. tGSH, total reduced glutathione; GSSG, oxidized glutathione disulfide.

## Abbreviations

AB	adaptive behaviors
ABC	Autism Behavior Checklist
AE	affective expression
ASD	Autism Spectrum Disorder
ATEC	Autism Treatment Evaluation Checklist
CARS	Children Autism Rating Scale
CBS	cystathionine-beta-synthase
CMB	characteristic motor behaviors
CVB	characteristic verbal behaviors
CVP	cognitive verbal/preverbal
DHFR	dihydrofolate reductase
EL	expressive language
FM	fine motor
GM	gross motor
GSH	reduced glutathione
GSSG	oxidized glutathione disulfide
Hcy	homocysteine
PB	problem behaviors
PEP-3	Psychoeducational Profile-third edition
PSC	personal self-care
RL	receptive language
SAM	S-adenosylmethionine
SR	social reciprocity
TEACCH	The Treatment and Education of Autistic and Related Communication Handicapped Children
VMI	visual motor imitation
